# High expression of *ETS2* predicts poor prognosis in acute myeloid leukemia and may guide treatment decisions

**DOI:** 10.1186/s12967-017-1260-2

**Published:** 2017-07-19

**Authors:** Lin Fu, Huaping Fu, Qingyun Wu, Yifan Pang, Keman Xu, Lei Zhou, Jianlin Qiao, Xiaoyan Ke, Kailin Xu, Jinlong Shi

**Affiliations:** 1 Department of Hematology and Lymphoma Research Center, Peking University, Third Hospital, Beijing, 100191 China; 2 Department of Hematology, The Affiliated Hospital of Xuzhou Medical University, Xuzhou, 221002 China; 3 Department of Hematology, Huaihe Hospital of Henan University, Kaifeng, 475000 China; 4 Department of Nuclear Medicine, Chinese PLA General Hospital, Beijing, 100853 China; 5 Department of Medicine, Wil-liam Beaumont Hospital, Royal Oak, MI 48073 USA; 6 Northeastern University, Boston, MA 02115 USA; 7 Department of Hematology, Chinese PLA General Hospital, Beijing, 100853 China; 8 Department of Biomedical Engineering, Chinese PLA General Hospital, Beijing, 100853 China; 9 Department of Medical Big Data, Chinese PLA General Hospital, Beijing, 100853 China

**Keywords:** *ETS2*, Prognosis, AML, Allogeneic HCT

## Abstract

**Background:**

*ETS2* is a downstream effector of the RAS/RAF/ERK pathway, which plays a critical role in the development of malignant tumor. However, the clinical impact of *ETS2* expression in AML remains unknown.

**Methods:**

In this study, we evaluated the prognostic significance of *ETS2* expression using two relatively large cohorts of AML patients.

**Results:**

In the first cohort, compared to low expression of *ETS2* (*ETS2* ^low^), high expression of *ETS2* (*ETS2* ^high^) showed significant shorter OS, EFS and RFS in the current treatments including the allogeneic HCT group (n = 72) and the chemotherapy group (n = 100). Notably, among *ETS2* ^high^ patients, those received allogeneic HCT had longer OS, EFS and RFS than those with chemotherapy alone (allogeneic HCT, n = 39 vs. chemotherapy, n = 47), but treatment modules play insignificant role in the survival of *ETS2* ^low^ patients (allogeneic HCT, n = 33 vs. chemotherapy, n = 53). Moreover, gene/microRNA expression data provides insights into the biological changes associated with varying *ETS2* expression levels in AML. The prognostic value of *ETS2* was further validated in the second AML cohort (n = 329).

**Conclusions:**

Our results indicate that *ETS2* ^high^ is a poor prognostic factor in AML and may guide treatment decisions towards allogeneic HCT.

**Electronic supplementary material:**

The online version of this article (doi:10.1186/s12967-017-1260-2) contains supplementary material, which is available to authorized users.

## Background

Acute myeloid leukemia (AML) represents a group of myeloid malignancies with remarkably heterogeneous outcomes [1]. Finding effective prognostic biomarkers has been one of the most urgent clinical needs and research hotspots. So far, a few prognosticators have been established, including mutations in *NPM1* and double *CEBPA* that are associated with favourable outcomes; whereas *FLT3*-*ITD* is associated with poor prognosis. High expression levels of *WT1* [2], *miR*-*155* [3, 4], *ERG* [5, 6], *BAALC* [6], and *MN1* [7] have also been shown to be poor prognostic factors in AML.

V-ets avian erythroblastosis virus E26 oncogene homolog 2 (*ETS2*), a downstream target for both the Ras/Raf/MAP kinase and phosphatidylinositol 3-kinase/Akt pathways. *ETS2* is one of the founder members of the E26 transformation-specific (*ETS*) family located on human chromosome 21 [8]. *ERG,* one of the classic prognostic markers in AML, also belongs to the *ETS* family. *ETS2* and *ERG* had been shown to be overexpressed in AML patients with complex karyotypes involving chromosome 21 [9]. Although *ETS2* was initially characterized as a proto-oncogene acute megakaryocytic leukemia (AMKL) [10], however, the clinical impact of *ETS2* expression in AML remains unknown.

In recent years, many studies suggest that *ETS2* exhibit both tumor-promoting and tumor-suppressive effects in malignancies. For example, *ETS2* has been found to be an oncogene in patients with AML [11], but it also has tumor-suppressive effects in non-small cell lung cancer [12]. Here, we demonstrate *ETS2* ^high^ as an adverse prognostic biomarker for AML based on analysis of two separate datasets and indicate *ETS2* ^high^ may guide treatment decisions towards allogeneic HCT; we also explore the distinctive gene/microRNA patterns associated with *ETS2* expression.

## Methods

### Patients

The first cohort was derived from The Cancer Genome Atlas (TCGA) dataset, including 200 clinically annotated adult de novo AML samples [13]. In this cohort, RNA sequencing for 179 samples and microRNA sequencing for 194 samples had been previously reported. Detailed descriptions of clinical and molecular characteristics were also provided. All these data were publicly accessible from the TCGA website. The study was approved by the human studies committee at Washington University with written informed consent obtained from all patients.

The second cohort was derived from a whole AML cohort (n = 329) diagnosed and collected at Erasmus University Medical Center (Rotterdam) between 1990 and 2008, approved by the institutional review boards at Weill Cornell Medical College and Erasmus University Center, and all subjects provided written informed consent in accordance with the Declaration of Helsinki. Microarray expression profiles were obtained by Affymetrix Human Genome 133 plus 2.0 and U133A Gene Chips from *GSE6891* data. All experiments’ design, quality control and data normalization were in line with the standard Affymetrix protocols. All clinical, cytogenetic and molecular information as well as microarray data of these patients were publicly accessible at the Gene Expression Omnibus (GSE6891, http://www.ncbi.nlm.nih.gov/geo) [14]. All patients were uniformly treated under the study protocols of Dutch-Belgian Cooperative Trial Group for Hematology Oncology (HOVON, details of therapeutic protocol available at http://www.hovon.nl).

### Statistical analyses

OS was defined as the time from the date of diagnosis to death due to any cause. EFS was defined as the time from the date of diagnosis to removal from the study due to the absence of complete remission, relapse or death. RFS was defined as the time from the date of diagnosis to removal from the study due to relapse.

Patients with higher than median *ETS2* expression values of all patients were classified as *ETS2* ^high^, and those with lower than median expression values were classified as *ETS2* ^low^. To investigate the associations between *ETS2* expression levels and clinical, molecular characteristics, the Fisher exact and Wilcoxon rank-sum tests were used for hypothesis testing with categorical and continuous variables, respectively. The associations between *ETS2* expression and the OS, EFS and RFS were analyzed by the Kaplan–Meier method and the log-rank test. Multivariate Cox proportional hazard models were employed to study the associations between *ETS2* expression levels and OS, EFS and RFS in the presence of other known risk factors. Student’s t test and multiple hypothesis correction (False Discovery Rate, FDR) was used to identify different gene/microRNA between *ETS2* ^high^ and *ETS2* ^low^ groups. The statistical cutoff values were an absolute fold-change (FC) ≥1.5 and an adjusted P value ≤0.05. All analyses were performed by the R 3.1.1 software packages.

## Results

### Expression of *ETS2* in AML patients and normal controls

**Fig. 1:**
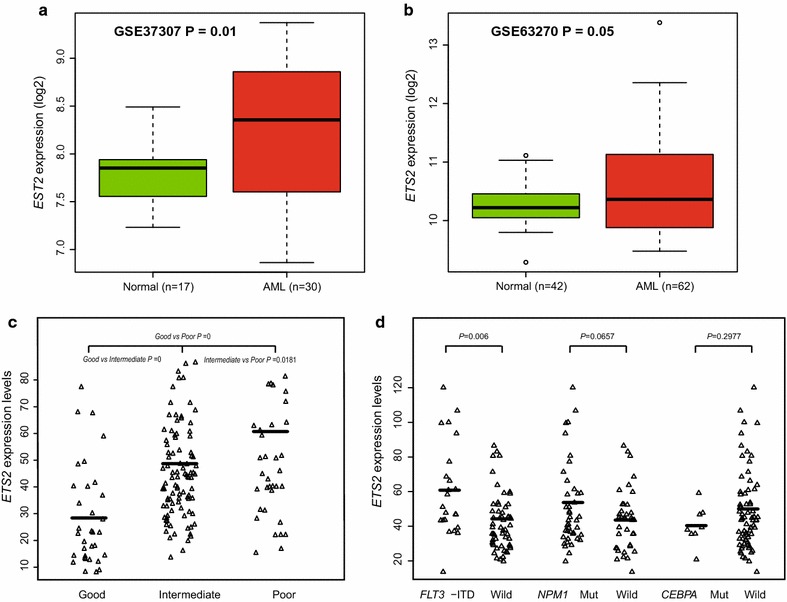
Differences in the expression of *ETS2* in AML. **a** AML-BM cases (n = 30) compared with NBM samples (n = 17), **b** AML-BM cases (n = 62) compared with NBM samples (n = 42), **c** relative expression of *ETS2* in the different NCCN-risk subgroup (good, intermediate and poor) of AML cases, **d** associations between *ETS2* expression and other classic prognostic biomarkers in AML cases (*FLT3*-ITD and the mutation of *NPM1* and *CEBPA*)

A microarray dataset of bone marrow (BM) samples was used for differential expression analysis, including 30 AML BM and 17 normal BM (NBM) samples (*GSE37307*, http://www.ncbi.nlm.nih.gov/geo), and 62 AML BM and 42 NBM samples (*GSE63270*, http://www.ncbi.nlm.nih.gov/geo). Higher expression of *ETS2* was shown significantly in AML BM than NBM (*P* = 0.01, Fig. 1a and *P* = 0.05, Fig. 1b).

### Relative expression of *ETS2* in different National Comprehensive Cancer Network (NCCN) risk subgroups

In the first cohort, *ETS2* showed averagely higher expression in the NCCN poor- and intermediate-risk patients than that in the good-risk group (good vs. intermediate *P* = 0, intermediate vs. poor *P* = 0.0181, and good vs. poor *P* = 0, respectively; Fig. 1c).

### Associations between *ETS2* expression and other classic prognostic biomarkers in AML

The first cohort were further divided into subgroups by the presence of *FLT3*-*ITD* and mutation status of *NPM1* and *CEBPA*. Levels of *ETS2* expression were compared among different subgroups. *ETS2* showed significantly higher expression in samples with *FLT3*-*ITD* compared than samples without *FLT3*-*ITD* (*P* = 0.006, Fig. 1d). No significant differences were revealed between *NPM1*-mutated and wild-type samples (*P* = 0.0657) or between *CEBPA*-mutated and wild-type samples (*P* = 0.2977, Fig. 1d).

### Differences in clinical and molecular characteristics between *ETS2*^high^ and *ETS2*^low^ groups

In the first cohort, *ETS2* ^high^ patients were more likely to be ≥60-year-old, and had higher WBC count, higher peripheral blood blasts, more diagnosed with M0, M1, M3, or M5 FAB subtypes, and more *FLT3*-*ITD* and *TP53* mutation (*P* = 0.004, *P* = 0.05, *P* = 0.01, *P* = 0.001, *P* = 0.005, *P* < 0.001, *P* = 0.04, *P* = 0.01, *P* = 0.016, respectively) comparing with *ETS2* ^low^ patients. No other associations between *ETS2* expression and other mutations were found. Additionally, *ETS2* ^high^ patients with AML were more likely to have a higher expression of *MN1*, *miR155HG* and *WT1* than *ETS2* ^low^ patients (*P* = 0.04, *P* < 0.001, and *P* = 0.009, respectively). See Table 1.

**Table 1:** Comparison of clinical and molecular characteristics of de novo AML patients according to ETS2

Variable	AML (TCGA dataset)		
	ETS2^high^ (n = 89)	ETS2^low^ (n = 90)	P
Median age, year (range)			0.01
Median	61	54.5	
Range	(18–88)	(22–82)	
Age group, n (%)			0.004
<60	38	58	
≥60	51	32	
WBC count, X10^9^/L			0.05
Median	33.2	12.2	
Range	0.6–297.4	0.4–202.7	
BM blasts (%)			0.6
Median	74	72	
Range	32–100	30–100	
PB blasts (%)			0.01
Median	49	25	
Range	0–98	0–97	
FAB subtype, no (%)			
M0	14	2	0.001
M1	30	14	0.005
M2	18	22	0.59
M3	0	16	<0.001
M4	16	19	0.6
M5	6	15	0.04
M6	1	1	1
Others	4	1	0.21
FLT3-ITD, n (%)			0.01
Present	25	12	
Absent	64	78	
NPM1 (no FLT3-ITD), n (%)			0.33
Mutated	12	17	
Wild-type	77	73	
CEBPA, n (%)			0.24
Single mutated	3	5	
Double mutated	1	4	
Wild-type	85	81	
MLL-PTD, n (%)			0.33
Mutated	6	3	
Wild-type	83	87	
IDH1, n (%)			0.58
Mutated	9	7	
Wild-type	80	83	
IDH2, n (%)			0.78
Mutated	9	8	
Wild-type	80	82	
RUNX1, n (%)			0.08
Mutated	12	5	
Wild-type	77	85	
DNMT3A, n (%)			0.21
R882 mutated	16	7	
Non-R822 mutated	9	11	
Wild-type	64	72	
TP53, n (%)			0.016
Mutated	12	3	
Wild-type	77	87	
ERG expression, n (%)			0.16
High	49	40	
Low	40	50	
BAALC expression, n (%)			0.07
High	50	39	
Low	39	51	
MN1 expression, n (%)			0.04
High	51	38	
Low	38	52	
miR155HG expression, n (%)			<0.001
High	56	33	
Low	33	57	
WT1 expression, n (%)			0.009
High	53	36	
Low	36	54	

High ERG, BAALC, MN1, miR155HG and WT1 expression were defined as an expression level above the median of all samples, respectively

*FAB* French–American–British classification, *FLT3-ITD* internal tandem duplication of the FLT3 gene, *MLL-PTD* partial tandem duplication of the MLL gene

### *ETS2*^high^ was associated with adverse outcomes

**Fig. 2:**
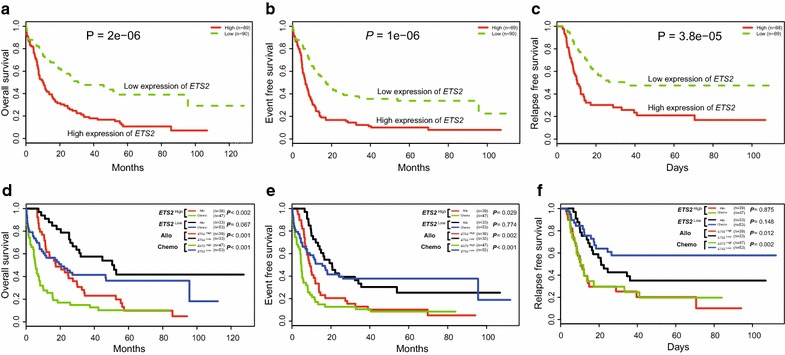
The prognostic value of *ETS2* expression in AML patients from TCGA data. **a** OS and **b** EFS and **c** RFS of the entire AML patients (n = 179). **d** OS and **e** EFS and **f** RFS of the AML patients of *ETS2* ^high^ group (n = 86), *ETS2* ^low^ group (n = 88), allogeneic HCT group (n = 72) and chemotherapy-only group (n = 100). *Allo* allogeneic HCT, *Chemo* chemotherapy

*ETS2* ^high^ patients had markedly shorter OS (Fig. 2a, *P* = 2e^−6^), EFS (Fig. 2b, *P* = 1e^−6^) and RFS (Fig. 2c, *P* = 3.8e^−5^) comparing with *ETS2* ^low^ patients. Associations between *ETS2* expression and prognostic significance within the allogeneic HCT group and chemotherapy group were also separately analyzed. Within the allogeneic HCT group (n = 72), significant differences were observed in OS (Fig. 2d, *P* < 0.001), EFS (Fig. 2e, *P* = 0.002) and RFS (Fig. 2f, *P* = 0.012) between the *ETS2* ^high^ and *ETS2* ^low^ patients. In the chemotherapy group (n = 99), *ETS2* ^high^ patients had significantly shorter OS (Fig. 2d, *P* < 0.001), EFS (Fig. 2e, *P* < 0.001) and RFS (Fig. 2f, *P* = 0.002) than *ETS2* ^low^ patients. Moreover, *ETS2* ^high^ patients who received allogeneic HCT had significantly longer OS and EFS than chemotherapy-only (OS, *P* < 0.002; EFS, *P* = 0.029, respectively), whereas treatment modules play insignificant role in the survival of *ETS2* ^low^ patients (allogeneic HCT vs. chemotherapy-only; OS, *P* = 0.067; EFS, *P* = 0.774; RFS, *P* = 0.148, respectively).

### *ETS2* expression was associated with shorter OS, EFS and RFS in multivariate analyses

To adjust for the impact of known clinical and molecular risk factors, we performed multivariate analyses to confirm the prognostic significance of *ETS2* expression (Table 2). In the multivariate models for OS, EFS and RFS, *ETS2* ^high^ had adverse impacts on OS (*P* = 0.002), EFS (*P* < 0.001) as well as RFS (*P* < 0.001). Age was the only other factor negatively correlated with OS (*P* < 0.001) and EFS (*P* < 0.001).

**Table 2:** Multivariable analysis with OS and EFS in the primary cohort of 179 AML patients (TCGA dataset)

Variables in final model by end point	HR	95% CI	*P* value
OS (all AML, n = 179)			
*ETS2* expression, high vs. low	1.79	1.23–2.59	0.002
Age, per 10-year increase	1.46	1.27–1.68	<0.001
*CEBPA* mutation vs. wild	1.75	0.85–3.58	0.13
*NPM1* mutation vs. wild	1.1	0.73–1.66	0.65
*FLT3*-ITD, presented vs. others	1.24	0.78–1.96	0.37
EFS (all AML, n = 179)			
*ETS2* expression, high vs. low	1.88	1.32–2.68	<0.001
Age, per 10-year increase	1.34	1.18–1.53	<0.001
*CEBPA* mutation vs. wild	1.2	0.92–3.57	0.08
*NPM1* mutation vs. wild	1.2	0.83–1.78	0.3
FLT3-ITD, presented vs. others	1.4	0.9–2.15	0.1
RFS (all AML, n = 177)			
*ETS2* expression, high vs. low	2.23	1.41–3.5	<0.001
Age, per 10-year increase	1.13	0.96–1.33	0.14
*CEBPA* mutation vs. wild	0.4	0.94–4.48	0.07
*NPM1* mutation vs. wild	0.25	0.81–2.15	0.26
FLT3-ITD, presented vs. others	1.47	0.86–2.53	0.16

*OS* overall survival, *EFS* event-free survival, *RFS* relapse-free survival, *HR* hazard ratio, *CI* confidence interval

### Associations between genome-wide gene-expression profiles and *ETS2* expression

**Fig. 3:**
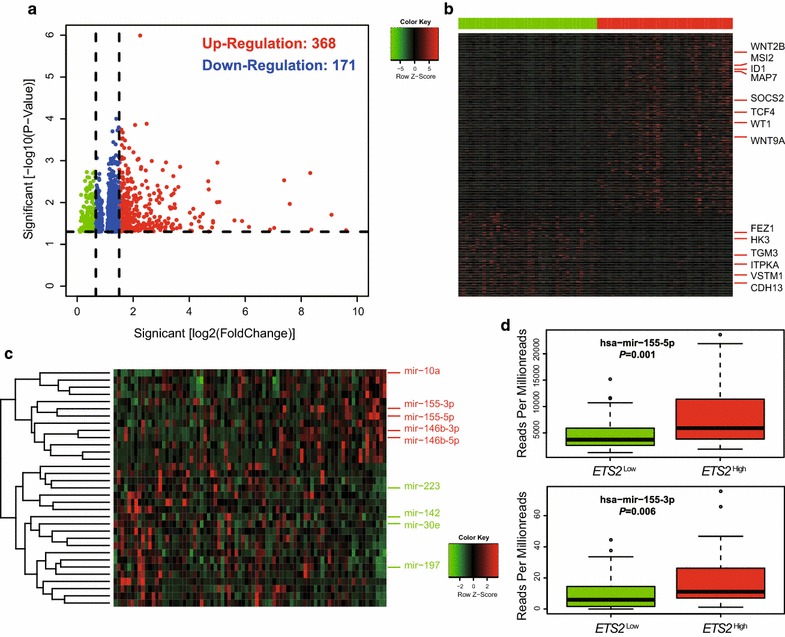
Genome-wide genes/microRNAs associated with *ETS2* expression. **a** Volcano plot of different gene-expression profiles between *ETS2* ^high^ and *ETS2* ^low^; *ETS2* ^high^ and *ETS2* ^low^ were marked by *red* and *green circles*, respectively. **b** Expression heatmap of *ETS2*-associated genes. **c** Expression heatmap of associated microRNAs. Patients are ordered from left to right by increasing *ETS2* expression. Expression values of the gene and microRNA probe sets are represented by *color*, with *green* indicating expression less than and *red* indicating expression greater than the value for the given gene and microRNA probe set. For the gene and miR-RNA expression heat map, up- and down-regulated genes and miR-RNAs mentioned in the text are indicated. **d** Boxplots of miR-155-5p and miR-155-3p expression associated with *ETS2* expression

To further assess the role of *ETS2* in AML, we derived *ETS2*-associated gene expression profiles by high throughput sequencing from TCGA data. We first identified 368 up-regulated and 171 down-regulated genes that were significantly associated with *ETS2* expression (*P* < 0.05, fold change = 1.5, Fig. 3a). With a more rigorous analysis (fold change = 2, and profiles without applicable values were all deleted), 359 genes were filtered out and the rest 180 genes were presented in an aberrant expression heat map (Fig. 3b).

Many genes known as unfavorable biomarkers were up-regulated, including leukemia-associated molecules, such as: (1) genes (*Wnt2B* and *Wnt9A*) of Wnt signaling pathway involved in leukemogenesis; (2) independent adverse prognostic factors in AML including *WT1*, *miR*-*155HG* [3, 4], *SOCS2* [15], *TCF4* [16], *MAP7* [17], *ID1* [18] and *MSI2* [19]. However, some tumor suppressors were down-regulated, such as: (1) *CDH13*, silenced by aberrant promoter methylation, similar silencing had been found to be involved in the pathogenesis in chronic myeloid leukemia (CML) [20]; (2) *VSTM1*, which had also been found down-regulated in bone marrow cells from leukemia patients and played an important role in the pathogenesis of leukemia [21]; (3) *CEBPA*-dependent *HK3* expression, its decrease promoted primary AML [22]; (4) *Fez1*, its absence impaired Cdk1/Cdc25C interaction during mitosis and in mouse models could predispose mice to cancer development [23]; (5) *TGM3*, a candidate tumor suppressor gene that contributed to human head and neck cancer [24]; (6) *ITPKA*, its down-regulation by early aberrant DNA methylation was also found in a mouse model of acute myeloid leukemia [25].

### Associations between genome-wide microRNA profiles and *ETS2* expression

An analysis of microRNA genome-wide profiles revealed 145 microRNAs that were strongly associated with *ETS2* expression (*P* < 0.05, Fig. 3c). *ETS2* ^high^ was positively correlated with levels of *miR*-*10a, miR*-*155, miR*-*146b* and *miR*-*1*. Notably, in the profiles we generated, *miR*-*155*-*3p* and *miR*-*155*-*5p* were up-regulated (Fig. 3d). In previous reports, these microRNAs were shown to have important tumor-promoting properties. For example, overexpression of *miR*-*10a* was associated with poor OS in AML patients [26]. Up-regulation of *miR*-*155* was an independent risk factor associated with an unfavorable clinical outcome in cytogenetically normal-AML (CN-AML) [3]. Knockdown of endogenous *miR*-*146b* would result in increased transcription of tumor suppressors and inhibition of cell proliferation in chronic lymphocytic leukemia (CLL) [27]. *MiR*-*1*-*2* modulation was vital for EVI1-associated tumor proliferation in acute myeloid leukemia [28].

*ETS2* ^high^ was negatively correlated with levels of *miR*-*223, miR*-*142, miR*-*30e* and *miR*-*197*. These microRNAs had been shown to exhibit tumor suppressive properties. Low *miR*-*223* expression was associated with worse outcome in AML [29]. *MiR*-*142*-*3p* was a key regulator of normal myeloid differentiation; its reduced expression was involved in the leukemogenesis of AML [30]. *MiR*-*30e* induced apoptosis and could sensitize cell lines to imatinib via regulation of the BCR-ABL protein [31]. *MiR*-*197* induced apoptosis and suppressed multiple myeloma by targeting MCL-1 [32].

### Association between *ETS2*^high^ and adverse outcomes was confirmed by the second cohort

**Fig. 4:**
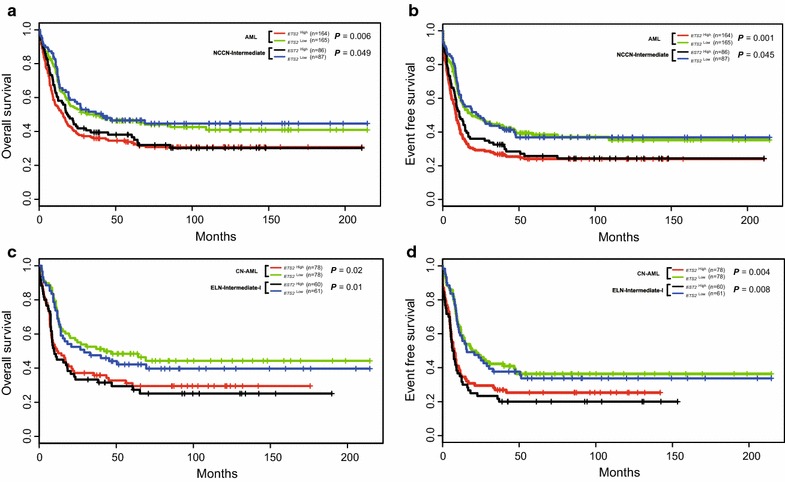
The prognostic value of *ETS2* expression in the second cohort. **a** OS and **b** EFS of 329 AML patients and the subgroup of 173 patients with NCCN intermediate-risk. **c** OS and **d** EFS of the 156 CN-AML patients and 121 AML patients in the ELN Intermediate-I category

We studied the second cohort of 329 previously untreated AML patients. Firstly, *ETS2* ^high^ AML patients (n = 164) had significantly shorter OS (*P* = 0.006, Fig. 4a) and EFS (*P* = 0.001, Fig. 4b) than *ETS2* ^low^ patients (n = 165). Secondly, in the NCCN intermediate-risk AML patients, *ETS2* ^high^ (n = 86) also had significantly shorter OS (*P* = 0.049, Fig. 4a) and EFS (*P* = 0.045, Fig. 4b) than *ETS2* ^low^ patients (n = 87). Thirdly, *ETS2* ^high^ CN-AML patients (n = 78) had significantly shorter OS (*P* = 0.02, Fig. 4c) and EFS (*P* = 0.004, Fig. 4d) than *ETS2* ^low^ patients (n = 78). Fourthly, for patients in the European Leukemia Net (ELN) Intermediate-I category, *ETS2* ^high^ (n = 60) also had significantly shorter OS (*P* = 0.01, Fig. 4c) and EFS (*P* = 0.008, Fig. 4d) than *ETS2* ^low^ patients (n = 61).

## Discussion

Identifying the prognostic factors for AML is important for the development of new targeted therapies and risk-stratified treatment strategies. Recent studies had shown that high expression of *ERG* and *ERG* amplification, the most frequent copy-number alteration (CNA), are all the worse prognostic markers in AML patients [5, 6, 33]. *ETS2,* one of the members of the *ETS* family as *ERG,* was previously characterized as a proto-oncogene in AMKL children that is Down-syndrome and non-Down-syndrome-related [10], but the expression and clinical prognosis of *ETS2* in AML remains unknown. Here, we have demonstrated the aberrant expression of *ETS2* in AML patients. First, we found that *ETS2* expression was up-regulated in AML cohorts and was overexpressed in the NCCN intermediate- and poor-risk groups of patients, compared to the good-risk group. These findings indicated that *ETS2* might promote leukemogenesis. We also found that *ETS2* showed higher expression in monocytes using publicly available expression data which suggest that *ETS2* might play an important role in the function of monocytes [34] (Additional file 1: Figure S1). Second, in the first cohort, our study demonstrated that *ETS2* ^high^ was associated with shorter OS and EFS. Notably, *ETS2* ^high^ patients had longer OS and EFS after receiving allogeneic HCT than chemotherapy-only, but similar differences between treatment modules were not observed in *ETS2* ^low^ patients. Its presence may direct treatment decisions towards allogeneic HCT.

To further confirm the prognostic significance of *ETS2*, we analyzed the second cohort of uniformly treated AML patients. *ETS2* ^high^ also acted as an independent poor prognostic factor in the entire cohort, NCCN Intermediate-risk subgroup, CN-AML subgroup, as well as the ELN Intermediate-I subgroup. The above results denoted that *ETS2* ^high^ was an independent, poor prognostic factor in AML. It could be employed to improve the risk stratification of ELN Intermediate-I category and NCCN Intermediate-Risk group.

Gene and microRNA-expression profiles derived from the first cohort gave us some insight regarding the role of *ETS2* in AML leukemogenesis. Tumor protein 53 (*TP53*) is one of the most frequently inactivated tumor suppressor genes in human cancer and its mutations predict a poor prognosis in patients with acute myeloid leukemia (AML) [35]. Recent studies have shown that mutations in the *TP53* (mTP53) protects *ETS2* from degradation and mTP53 disrupts ETS family target gene regulation, promoting cancer [36]. In our study, we found that *ETS2* ^high^ was associated with mTP53.

The expression of *miR*-*155* has been found to be independently associated with poor clinical outcome in AML [3, 4]. In addition, we found that *ETS2* ^high^ was associated with over-expression of *miR*-*155HG*, *miR*-*155*-*3p* and *miR*-*155*-*5p*. This result is in accordance with recent studies which have found that *ETS2* is an important transcription factor regulating *miR*-*155* [37].

## Conclusions

In summary, *ETS2* ^high^ is an independent poor prognostic factor in AML patients and its presence should favor allogeneic HCT over chemotherapy-only in AML. In AML patients, distinctive gene/microRNA expression profiles associated with *ETS2* expression may explain the role of *ETS2* in the leukemogenic process.

## Additional file

**Additional file 1.** The hierarchical differentiation tree of relationship between ETS2 expression level and hematopoietic cell differentiation.

## Abbreviations

*ETS*: E26 transformation-specific
*ETS2*: V-ets avian erythroblastosis virus E26 oncogene homolog 2
AML: acute myeloid leukemia
AMKL: acute megakaryocytic leukemia
OS: overall survival
EFS: event-free survival
RFS: relapse-free survival
HCT: hematopoietic cell transplantation
FAB: French–American–British classification
FLT3-ITD: internal tandem duplication of the FLT3 gene
MLL-PTD: partial tandem duplication of the MLL gene
HR: hazard ratio
CI: confidence interval
