# Supplementary material for: High expression of ETS2 predicts poor prognosis in acute myeloid leukemia and may guide treatment decisions
Source: J Transl Med. 2017 Jul 19;15:159. doi: 10.1186/s12967-017-1260-2 (PMC5518161; doi:10.1186/s12967-017-1260-2)
Supplement: Supplementary file 1 — Additional file 1. The hierarchical differentiation tree of relationship between ETS2 expression level and hematopoietic cell differentiation. [file 12967_2017_1260_MOESM1_ESM.pdf]

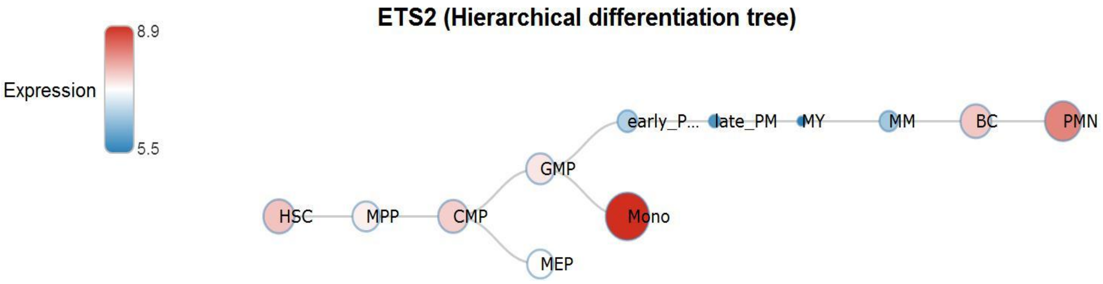

Figure S1. The hierarchical differentiation tree of relationship between ETS2 expression level and hematopoietic cell differentiation. HSC: Hematopoietic stem cell; MPP: Multipotential progenitors; CMP: Common myeloid progenitor cell; GMP: Granulocyte monocyte progenitors; MEP: Megakaryocyte-erythroid progenitor cell; early-PM: Early Promyelocyte; late-PM: Late Promyelocyte; MY: Myelocyte; MM: Metamyelocytes; BC: Band cell; PMN: Polymorphonuclear cells; Mono: Monocytes.
